# Identification of odorant binding proteins and chemosensory proteins in *Microplitis mediator* as well as functional characterization of chemosensory protein 3

**DOI:** 10.1371/journal.pone.0180775

**Published:** 2017-07-21

**Authors:** Yong Peng, Shan-Ning Wang, Ke-Ming Li, Jing-Tao Liu, Yao Zheng, Shuang Shan, Ye-Qing Yang, Rui-Jun Li, Yong-Jun Zhang, Yu-Yuan Guo

**Affiliations:** 1 College of Plant Protection, Agricultural University of Hebei, Baoding, China; 2 State Key Laboratory for Biology of Plant Diseases and Insect Pests, Institute of Plant Protection, Chinese Academy of Agricultural Sciences, Beijing, China; 3 Institute of Banana and Plantain, Chinese Academy of Tropical Agricultural Sciences, Haikou, China; 4 College of Plant Protection, China Agricultural University, Beijing, China; Universita degli Studi della Basilicata, ITALY

## Abstract

Odorant binding proteins (OBPs) and chemosensory proteins (CSPs) play important roles in transporting semiochemicals through the sensillar lymph to olfactory receptors in insect antennae. In the present study, twenty OBPs and three CSPs were identified from the antennal transcriptome of *Microplitis mediator*. Ten OBPs (*MmedOBP11–20*) and two CSPs (*MmedCSP2–3*) were newly identified. The expression patterns of these new genes in olfactory and non-olfactory tissues were investigated by real-time quantitative PCR (qPCR) measurement. The results indicated that *MmedOBP14*, *MmedOBP18*, *MmedCSP2* and *MmedCSP3* were primarily expressed in antennae suggesting potential olfactory roles in *M*. *mediator*. However, other genes including *MmedOBP11*–*13*, *15–17*, *19*–*20* appeared to be expressed at higher levels in body parts than in antennae. Focusing on the functional characterization of MmedCSP3, immunocytochemistry and fluorescent competitive binding assays were conducted indoors. It was found that MmedCSP3 was specifically located in the sensillum lymph of olfactory sensilla basiconca type 2. The recombinant MmedCSP3 could bind several types of host insects odors and plant volatiles. Interestingly, three sex pheromone components of Noctuidae insects, *cis*-11-hexadecenyl aldehyde (*Z*11-16: Ald), *cis*-11-hexadecanol (*Z*11-16: OH), and *trans*-11-tetradecenyl acetate (*E*11-14: Ac), showed high binding affinities (Ki = 17.24–18.77 μM). The MmedCSP3 may be involved in locating host insects. Our data provide a base for further investigating the physiological roles of OBPs and CSPs in *M*. *mediator*, and extend the function of MmedCSP3 in chemoreception of *M*. *mediator*.

## Introduction

In insects, behaviors of host identification, mating-partners, and locating oviposition sites are regulated largely by volatile chemical cues [1, 2]. These odor molecules are usually perceived by chemosensory sensilla located on their antennae [3]. In insect olfaction model, odorants enter the chemosensory sensilla through cuticular pores, dissolved in the sensillum lymph and are captured by soluble carrier proteins, then activate sensory neurons [4, 5]. Two major classes of soluble proteins, odorant binding proteins (OBPs) and chemosensory proteins (CSPs) play essential roles in transferring semiochemicals to the membrane-bonded receptors, and may contribute to the activation of olfactory receptor neurons [6, 7].

OBPs and CSPs were synthesized in non-neuronal support cells and secreted in the lymph of chemosensilla at extraordinarily high concentrations [8]. Both OBPs and CSPs are small (12–18 kDa) and soluble proteins which have capacities to bind reversibly small molecules [9, 10]. OBPs were usually classified in different type depend on the motif of conserved cysteines, and subdivide as typical OBPs, “Plus-C” OBPs, “Minus-C” OBPs, Dimer OBPs and Atypical OBPs. Normal OBP include pheromone binding proteins (PBPs), general odorant binding proteins (GOBPs) and antennal specific proteins (ASPs) or antennal-binding protein x (ABPx) [4, 11]. The first OBP was identified from *Antheraea polyphemus* and named ApolPBP1 [12]. Since then lots of OBPs and CSPs were identified and characterized in insect species [13, 14]. Generally, most of OBPs are considered to be antenna-specific, whereas CSPs are more widely distributed in different tissues of the insect body suggesting their multi-roles [15–17].

*Microplitis mediator* (Hymenoptera: Braconidae) is a polyphagous solitary larval endoparasitoid that parasitizes approximately 40 species of Lepidoptera insects [18]. Several odor carrier proteins including ten OBPs (MmdeOBP1–10) and one CSP (MmedCSP1) of *M*. *mediator* were identified in our previous work [19, 20]. The binding characteristics of these proteins were also investigated by using fluorophore displacement assays [20, 21]. However, some plant volatiles, such as the (*Z*)-3-hexenyl acetate and (*E*)-2-hexenal, which could elicit electrophysiological and behavioral responses of *M*. *mediator* showed no any binding affinity to above mentioned MmedOBPs and MmedCSPs [22, 23]. Thus, we suspected that there may be more OBPs and CSPs unexplored in the antennae of *M*. *mediator*, which could bind more potential semiochemicals. Encouragingly, the development of new sequencing technology provides a strong support for the discovery of these genes.

In the current study, ten OBPs (*MmedOBP11–20*) and two CSPs (*MmedCSP2–3*) were newly identified from the antennal transcriptome of *M*. *mediator*. The tissue- and sex- specific expression profile was investigated by using quantitative real-time PCR (qPCR). Focusing on the functional characterization of MmedCSP3, immunocytochemistry and fluorescent competitive binding assays were performed indoors. Our results will provide a theoretical basis for further investigating the physiological roles of OBPs and CSPs in *M*. *mediator*.

## Materials and methods

### Insects

A colony of *M*. *mediator* was obtained from the Institute of Plant Protection, Hebei Academy of Agriculture and Forestry, China. Emerged adult parasitoids were fed with a 10% honey solution in a growth chamber under the conditions of 28 ± 1°C, 75% relative humidity, and a 16: 8 (Light: Dark) photoperiod [24]. Female antennae, male antennae, heads (without antennae), thoraxes, abdomens, legs and wings from two to three days old adult wasps were excised and immediately frozen in liquid nitrogen, then stored at –80°C before qPCR analysis.

### Total RNA extraction

Total RNA was extracted from different tissue samples by using Trizol reagent (Invitrogen Carlsbad, CA, USA). The integrity of extracted RNA was checked by using 1.2% agarose gel electrophoresis, and quantified using a ND-2000 spectrophotometer (NanoDrop, Wilmington, DE, USA) at OD_260_ nm. The total RNA was treated with RQ1 RNase-Free DNase (Promega, Madison, USA) at 37°C for 30 min to remove residual DNA. The cDNAs were synthesized using the Fast Quant RT Kit (TianGen, Beijing, China).

### Identification of OBPs and CSPs

The tBLASTn program was utilized with available sequences of OBPs and CSPs from Hymenoptera species as “query” sequences to identify candidate unigenes that encode OBPs and CSPs in *M*. *mediator* [25]. All candidate transcripts were manually checked by using BLASTx program of the NCBI website (http://blast.ncbi.nlm.nih.gov/Blast.cgi) [26]. Gene-specific primers were designed with the Beacon Designer 7 (PREMIER Bio-soft International) to amplify the sequences of *MmedOBP11–20* and *MmedCSP2–3* (Table 1). Each reaction (25 μl volume) containing 200 ng of cDNA from different tissues was used as a template. The cycling parameters were: 95°C for 3 min; with 35 cycles as follows: 94°C for 45 sec, 58°C for 1 min, 72°C for 1 min; and a final extension step of 10 min at 72°C. The PCR product was gel-purified and sub-cloned into the pEasy-T3 vector (TransGen, Beijing, China) and then sequencing validation was performed.

**Table 1:** Primers used in this study.

Primer name	Forward (5'—3')	Reverse (5'—3')
**For sequencing**		
*MmedOBP11*	ACATGGGGACTCAGTAATTTGTAAGC	CATAATTTATGACATCATTGCATAGTCG
*MmedOBP12*	CTGAAGGTAATAAGACAAGATATGGC	TTAATTATATATTTTATCCTGATGCACATCC
*MmedOBP13*	ATGAAAATAATCGCTGTTATATTCGCTG	TCAGGCGCTCCTTGGAGATGGC
*MmedOBP14*	ATGAAGGGTGTAAAAAGTATCCCTTTAA	TTATGGATAAAAGTAACTCGGTGCATCC
*MmedOBP15*	ATGAAGAATATTTTATTAGGGATTTGTA	TCAAAATAAGTAATACGTCTTTGGTGATAC
*MmedOBP16*	ATGAAATTATTCGCTGTATTATTCGCC	TTACGAGCTCTTTTCAGTAATTATCTTAGTG
*MmedOBP17*	GTGCTGATTTGTATCTACACCACAACC	GACAGAATTTCGTAGTCACATGAAG
*MmedOBP18*	TTCATCATTGCTATACTAGCGTTAGAA	ACATGTCCGTTAAACTCAGTAGTCG
*MmedOBP19*	CCTATCTGATAGTAAAACTTCAATC	AAGTTTAATTCCACCGGATAC
*MmedOBP20*	ATGCAGATGCAAGTGAATGCTGACA	TTATACAAATGACACGCTCTTGAGCAC
*MmedCSP2*	CATCAATGCTAAATAGACTGGCAC	CTTCAAATTTTACAAACTGCCGAG
*MmedCSP3*	CTATAAGGAATATTTTTAACCAGACAAG	ACTAATTGGTGATTGACAGATTTGC
**For qPCR**		
*MmedOBP11*	GAAGATAGGCGACGAAGTGC	ATTATCATGGTGGCGGTCTC
*MmedOBP12*	TCATGGGGGATTCAAAGTTC	ACCTTTTCCCAGCAGACAGA
*MmedOBP13*	ACCTTTTCCCAGCAGACAGA	GCGTGACACTTAGCCATCAA
*MmedOBP14*	TGCAAGAGTGACGAAAGTTTTT	TATCGCTGCACGAGAACAAA
*MmedOBP15*	CCCAGTTTCGTGTCTGATGA	GACTCTGGCCACTGTCCATT
*MmedOBP16*	GAATGGTTGATGGACGACCT	CTTGCGAGCTTTCTCTTCGT
*MmedOBP17*	CGACCTTAAGCTTCGGTGTT	GCCATCCTCTTGCGAGTTAT
*MmedOBP18*	TTCCGATGCTGAATCAAGTG	CCTCATTGTCAGCGTTCTCA
*MmedOBP19*	TGACTGCAGACGGTTCTTTG	GCCACATGATCTGATTGCAC
*MmedOBP20*	GCCTTTAATGCCTGTCGGTA	GCCTTTAATGCCTGTCGGTA
*MmedCSP2*	TCTCTGCGGAGGCTGTAAAT	GCAGGGTTTCTCGTCCATTA
*MmedCSP3*	TAAAGGGCCATGTGGAGAAG	GTGTACCAGTCGGCGATTTT
*β-actin*	CCGTGCTTTCTCTCTACGCT	AGGTAGAGCGTAACCCTCGT
**For recombinant expression**		
*MmedCSP3*	TACTCACCATGGGAGACGAACTTTATTCAGATAAATATG	TACTCACTCGAGTCATTTCCTGCTATCGCCGA

Note: the restriction enzyme sites used for recombinant expression are underlined.

### Sequence analysis

The putative N-terminal signal peptides of OBPs and CSPs were predicted using the SignalP 4.0 server (http://www.cbs.dtu.dk/services/SignalP/). The open reading frames (ORFs) and the associated amino acid sequences were determined by using GeneDoc 2.7.0 software. The phylogenetic tree for OBP and CSP was constructed based on MmedOBP/CSP sequences and orthologues in other Hymenoptera species [15, 27–29]. Amino acid sequences were aligned using the program Clustal X 2.0 [30]. A neighbor-joining tree was constructed using the MEGA 5.0 program [31] with a p-distance model and a pairwise deletion of gaps. Bootstrapping was performed by the re-sampling amino acid positions of 1000 replicates.

### qPCR analysis

The relative transcript abundance of *MmedOBP* and *MmedCSP* genes in different tissue samples were evaluated by using qPCR measurement on an ABI Prism 7500 Fast Detection System (Applied Biosystems, Carlsbad, CA). The reference gene β-actin (GenBank accession number: KC193266) of *M*. *mediator* was used as the endogenous control to normalize the target gene expression and correct for any sample-to-sample variation. The primers (Table 1) of the target and reference genes were designed by Primer 3.0 program. The specificity of each primer set was validated by melt-curve analysis, and the efficiency was calculated by analyzing standard curves with a five-fold cDNA dilution series. Each qPCR reaction was conducted in a 20 μl mixture containing 10 μl of 2 × SuperReal PreMix Plus (TianGen, Beijing, China), 0.6 μl of each primer (10 μM), 0.4 μl of 50 × Rox Reference Dye, 1 μl of sample cDNA, 7.4 μl of sterilized H_2_O. The qPCR cycling parameters consisted of 95°C for 15 min, followed by 40 cycles of 95°C for 10 sec and 60°C for 30 sec, and melt curves stages at 95°C for 15 sec, 60°C for 1 min, and 95°C for 15 sec. The experiments for the test samples, endogenous control and negative control were performed in triplicate to ensure reproducibility. Relative quantification was performed using the comparative 2^-ΔΔCt^ method [32]. All of the data were normalized to endogenous β-actin levels from the same tissue samples. The cycle threshold (CT) values of OBPs and CSPs in different tissues are listed in S1 Table.

### Expression and purification of recombinant MmedCSP3

*MmedCSP3* was PCR-amplified using gene-specific primers (Table 1). The sample cDNA of the antennae was used as the template. The PCR product was first cloned into a pEASY-T3 Vector (Takara, Dalian, China) and then excised and cloned into an expression vector pET-30a (+) for expression in prokaryotic BL21 (DE3) cells (Takara, Dalian, China). The transformation of the strain with pET-30a (+) / MmedCSP3 was incubated in 500 mL of LB medium with 100 μg / ml kanamycin at 37°C. When the OD of the culture reached 0.4–0.6, the protein was induced with 1 mM isopropylthio-β-galactoside (IPTG) at 16°C and vibrated for 16 h at 200 rpm. The cultures were harvested by centrifugation at 12000 × g for 25 min at 4°C. The supernatant was obtained by sonication, purified by Ni ion affinity chromatography (ÄKTA avant 25, GE Healthcare, USA). Soon after the His-tag was removed with recombinant enterokinase (Novoprotein, Shanghai, China), followed by a second purification mentioned above. A 15% sodium dodecyl sulfate polyacrylamide gel electrophoresis (SDS-PAGE) analysis was conducted to check the size and purity of recombinant MmedCSP3.

### Western blot analysis

Polyclonal antiserum against recombinant MmedCSP3 was obtained by injecting robust adult rabbits subcutaneously and intramuscularly with the highly purified recombinant MmedCSP3 proteins. The protein was emulsified with an equal volume of Freund’s complete adjuvant (Sigma, St. Louis, MO, USA) for the first injection (500 μg of recombinant protein) and incomplete adjuvant for the three additional injections (300 μg each time). The interval between each injection was approximately half a month, and rabbit blood was collected seven days after the last injection and centrifuged at 6000 rpm for 20 min. The serum was further purified using a MAb Trap kit (GE Healthcare, Milwaukee, WI, USA) following the manufacturer’s instructions. The rabbits were maintained in large cages at room temperature, and all of the operations were performed according to ethical guidelines to minimize the pain and discomfort of the animals.

The purified recombinant MmedCSP3 were separated by 15% SDS-PAGE and then transferred to a polyvinylidene fluoride (Millipore, Carrigtwohill, Ireland) membrane. Subsequently, the membrane was blocked with 5% skimmed milk in phosphate-buffered saline (PBS) containing 0.05% Tween-20 (PBST) overnight at 4°C. After washing thrice with PBST (10 min each time), the blocked membrane was incubated with the purified rabbit anti-MmedCSP3 antiserum (dilution 1: 5,000) for 1 h at room temperature. After washings thrice with PBST, the membrane was incubated with goat anti-rabbit IgG horseradish peroxidase (HRP) conjugate and HRP-streptavidin complex (Promega, Madison, WI, USA) at a dilution of 1: 10,000 for 1 h. The membrane was then incubated with the Easy-See Western Blot kit (TransGen Biotech, Beijing, China), and exposed by Image-Quant LAS 4000 mini (GE Healthcare Bio-Sciences AB, Uppsala, Sweden).

### Immunocytochemical localization

Male or female antennae of two- or three-day-old adult wasps were fixed in a mixture of paraformaldehyde (4%) and glutaraldehyde (2%) in 0.1 M PBS (pH 7.4) at room temperature for 24 h, dehydrated in an ethanol series, and embedded in LR white resin (Taab, Aldermaston, Berks, UK) for polymerization at 60°C. Ultrathin sections (60–80 nm) were cut using a diamond knife on a Reichert Ultracut ultramicrotome (Reichert Company, Vienna, Austria). For immunocytochemical assay, the grids were subsequently floated in 25 μl droplets of PBSG (PBS containing 50 mM glycine) and PBGT (PBS containing 0.2% gelatin, 1% bovine serum albumin, and 0.02% Tween-20) and incubated with purified rabbit anti-MmedCSP3 antiserum (dilution 1: 2,000) at 4°C overnight. After washing six times with PBGT, the sections were incubated with secondary antibody (anti-rabbit IgG) coupled with 10 nm colloidal gold granules (Sigma, St. Louis, MO, USA) at a dilution of 1: 20 at room temperature for 90 min. Before being observed with a Hitachi H-7500 TEM (Hitachi Ltd., Tokyo, Japan), the sections were subjected to optional silver intensification for 15 min and stained with 2% uranyl acetate to increase the contrast. The serum supernatant from an un-injected healthy rabbit at the same dilution rate acted as the negative control. Three male and female adult antennae were respectively used in immunocytochemical assays.

### Fluorescence binding test

The binding abilities of MmedCSP3 to 102 candidate chemicals were measured on a fluorescence spectrometer (F-380, Tianjin, China) in a 1 cm light path quartz cuvette with 10 nm slits for both excitation and emission. The 4, 4’-dianilino-1,1’-binaphthyl-5,5’-sulfonic acid (bis-ANS, Sigma, Riedstr, Steinheim, Germany) as fluorescent probe was excited at 365 nm and emission spectra were recorded from 435 to 620 nm. The bis-ANS and all compounds were dissolved in HPLC purity-grade methanol with a concentration of 1 mM. 2 μM solution of the protein in 50 mM Tris-HCl (pH 7.4) was titrated with aliquots of bis-ANS prepared in advance to final concentrations of 2–16 μM to measure the affinity of bis-ANS to the protein. The affinities of other ligands were measured in competitive binding assays with the protein and bis-ANS at 2 μM in the presence of each competitor at 2–40 μM.

The fluorescence intensities at the maximum fluorescence emission between 435 to 620 nm were plotted against the free ligand concentration to determine the binding constants. The bound chemical was evaluated based on its fluorescence intensity with the assumption that the protein was 100% active with a stoichiometry of 1: 1 (protein: ligand) saturation. The binding curves were linearized using a Scatchard plot, and the dissociation constants of the competitors were calculated from the corresponding IC_50_ values based on the following equation: K_i_ = [IC_50_] / (1 + [bis-ANS] / K_bis-ANS_), where [bis-ANS] is the free concentration of bis-ANS and K_bis-ANS_ is the dissociation constant of the complex protein / bis-ANS [33].

### Statistical analysis

Data analysis was performed using SPSS Statistics 18.0 software (SPSS Inc., Chicago, IL, USA). ANOVA and Tukey HSD new multiple range test (*P* < 0.05) were used to compare the expression of each target gene among various tissues.

## Results

### Identification of OBPs and CSPs

**Fig 1:**
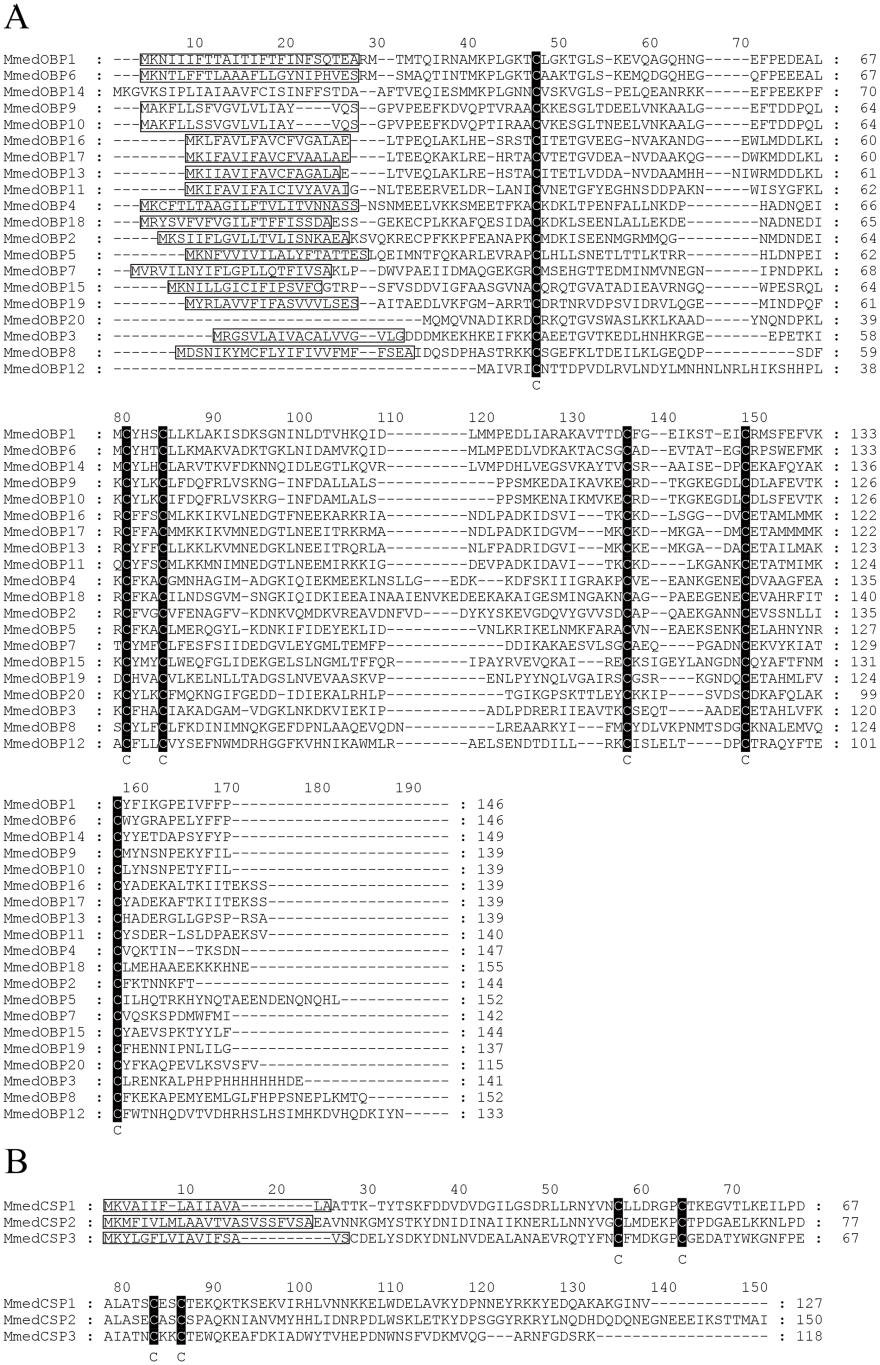
Sequence alignment of all identified OBPs and CSPs in *M*. *mediator*. A: Sequence alignment of OBPs, six conserved cysteine residues are marked in black; B: Sequence alignment of CSPs, four conserved cysteine residues are marked in black. Predicted signal peptides are boxed in the figure.

**Fig 2:**
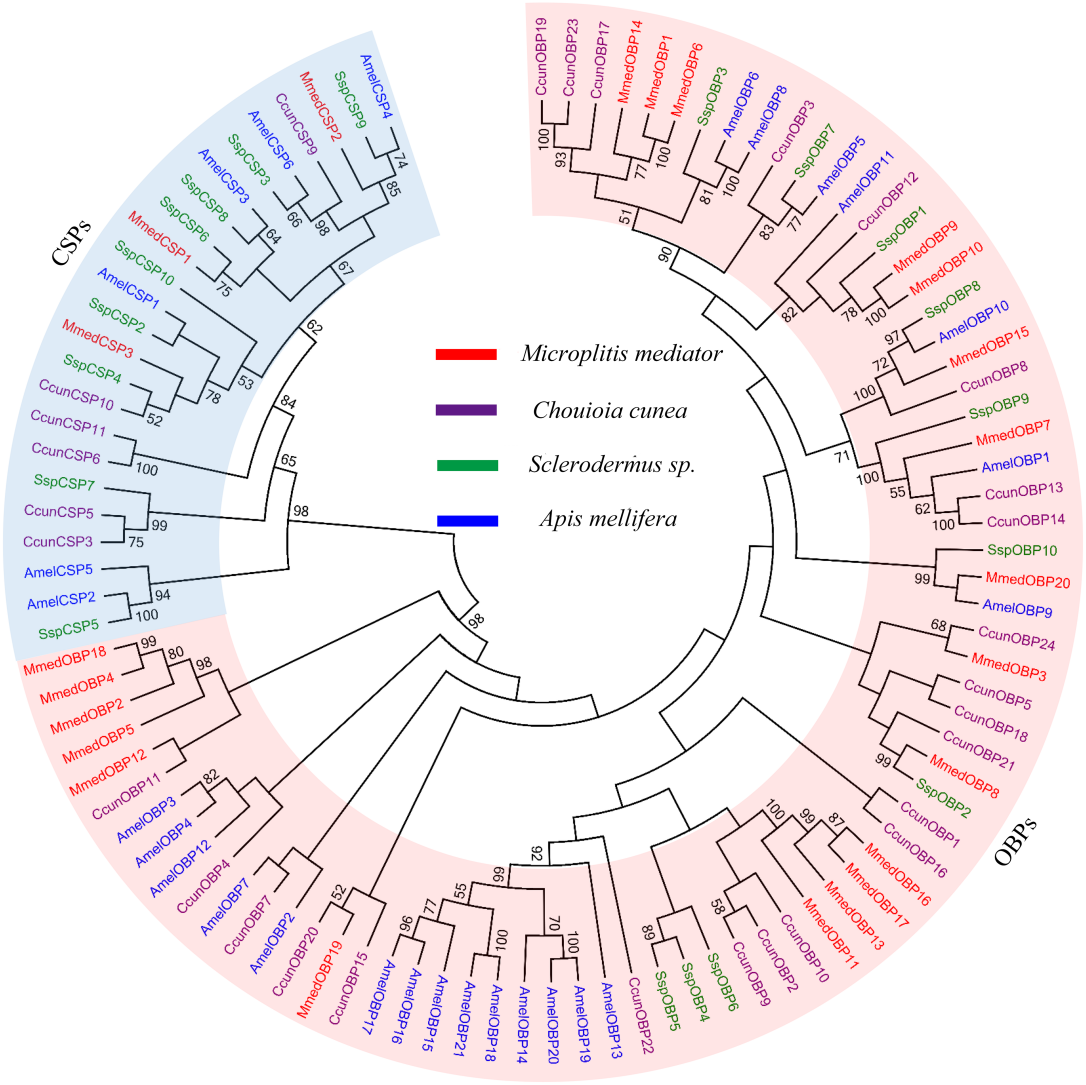
Phylogenetic tree of OBPs and CSPs from Hymenoptera species. Mmed (red): *M*. *mediator*; Ccun (purple): *Chouioia cunea*; Ssp (green): *Sclerodermus sp*.; Amel (blue): *Apis mellifera*.

In the present study, a total of twenty *MmedOBPs* and three *MmedCSPs* were identified from antennal transcriptome of *M*. *mediator*. The BLASTx results indicated that ten OBPs and two CSPs were newly identified (Table 2), which were named as *MmedOBP11–20* and *MmedCSP2–3*, respectively, based on our previous work [20]. Besides *MmedOBP12*, *MmedOBP14* and *MmedOBP20*, all other proteins had intact ORFs and encoded polypeptides with signal peptide (Fig 1A). All the twenty MmedOBPs had six conserved cysteine residues, while three MmedCSPs had four conserved cysteine residues (Fig 1B). After sequencing, all sequences of newly identified *MmedOPBs* and *MmedCSPs* were deposited in GenBank with the accession numbers listed in Table 2. The phylogenetic tree showed that OBPs and CSPs almost clustered in two distinct groups. Fourteen MmedOBPs are clustered in four species-specific clades, whereas six MmedOBPs were significantly divergent from each other and clustered together with their orthologs in other species (Fig 2). Each of the MmedCSPs was clustered together with the CSPs from other Hymenoptera species.

**Table 2:** OBPs and CSPs newly identified in antennae of *M*. *mediator*.

Genes	ORF	Length (aa)	Accession number	Signal Peptide (aa)	Best BLASTx hit			
					Species	Protein ID	E-value	Identity
*MmedOBP11*	423	141	KU342573	1–17	*Microplitis demolitor*	XP_008548248.1	2e-41	55
*MmedOBP12*	402	134	KU342574	ND	*M*. *demolitor*	AJM71483.1	1e-17	31
*MmedOBP13*	420	140	KU342575	1–17	*M*. *demolitor*	XP_008548248.1	1e-59	69
*MmedOBP14*	450	150	KU342576	ND	*M*. *demolitor*	XP_008543322.1	2e-29	40
*MmedOBP15*	435	145	KU342577	1–17	*M*. *demolitor*	XM_008561265.1	7e-87	98
*MmedOBP16*	420	140	KU342578	1–17	*M*. *demolitor*	XP_008548247.1	3e-69	83
*MmedOBP17*	420	140	KU342579	1–17	*M*. *demolitor*	XP_008548248.1	8e-73	93
*MmedOBP18*	468	156	KU342580	1–21	*M*. *demolitor*	XP_008543433.1	5e-34	49
*MmedOBP19*	414	138	KU342581	1–19	*Fopius arisanus*	XP_011312148.1	8e-28	42
*MmedOBP20*	348	116	KU342582	ND	*M*. *demolitor*	XP_008546277.1	3e-78	100
*MmedCSP2*	453	151	KU342583	1–23	*Camponotus japonicus*	BAS29778.1	1e-42	67
*MmedCSP3*	357	119	KU342584	1–18	*Apis cerana*	ACI03402.1	2e-27	45

Note: aa, amino acids; ND, not detected.

### Tissue expression profiles of *MmedOBPs* and *MmedCSPs*

**Fig 3:**
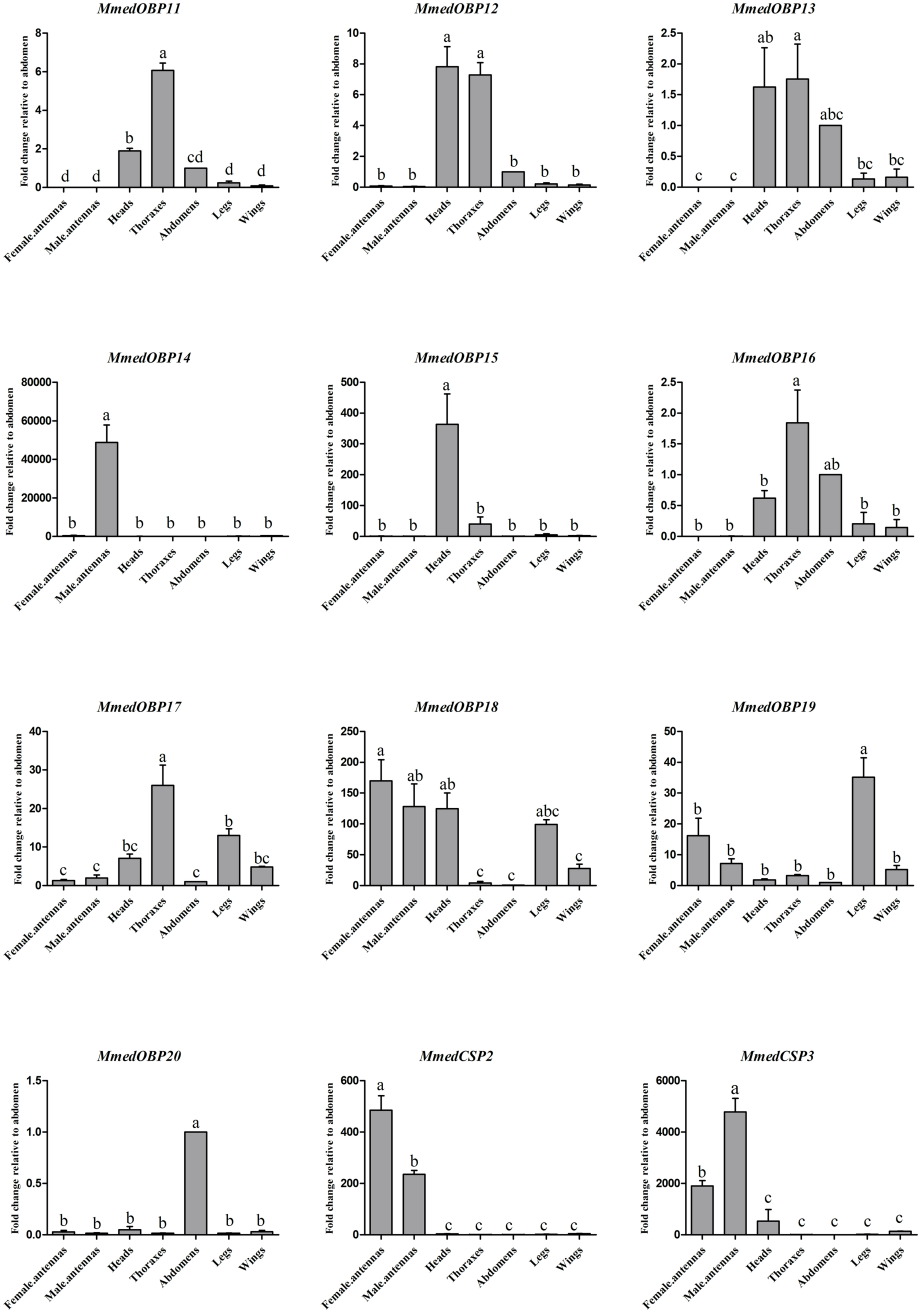
qPCR analysis of *MmedOBPs* and *MmedCSPs* expression in different tissues. The error bars represent standard error and the different small letters above each bar indicate significant differences in transcript abundances (*P* < 0.05).

The qPCR results showed that only two OBPs, *MmedOBP14* and *MmedOBP18*, were highly expressed in antennae (Fig 3). *MmedOBP14* was specific expressed in antennae of male wasps, while *MmedOBP18* had slightly high expressions in antennae of female adults. Interestingly, some *MmedOBPs* were mainly expressed in non-olfactory tissues. *MmedOBP15* was mainly expressed in the heads of wasps, *MmedOBP19* was primarily expressed in the legs rather than in the antennae, *MmedOBP20* was only detected in the abdomen, *MmedOBP12* and *MmedOBP13* were primarily expressed in the heads and thoraxes; while three OBPs (*MmedOBP11*, *16*, *17*) were highly enriched in the thoraxes. Two *MmedCSPs* were mainly expressed in the antennae of adult wasps. The transcript level of *MmedCSP2* was higher in females than in males, while *MmedCSP3* showed high expressions in antennae of both male and female wasps.

### Location of MmedCSP3 in antennae

**Fig 4:**
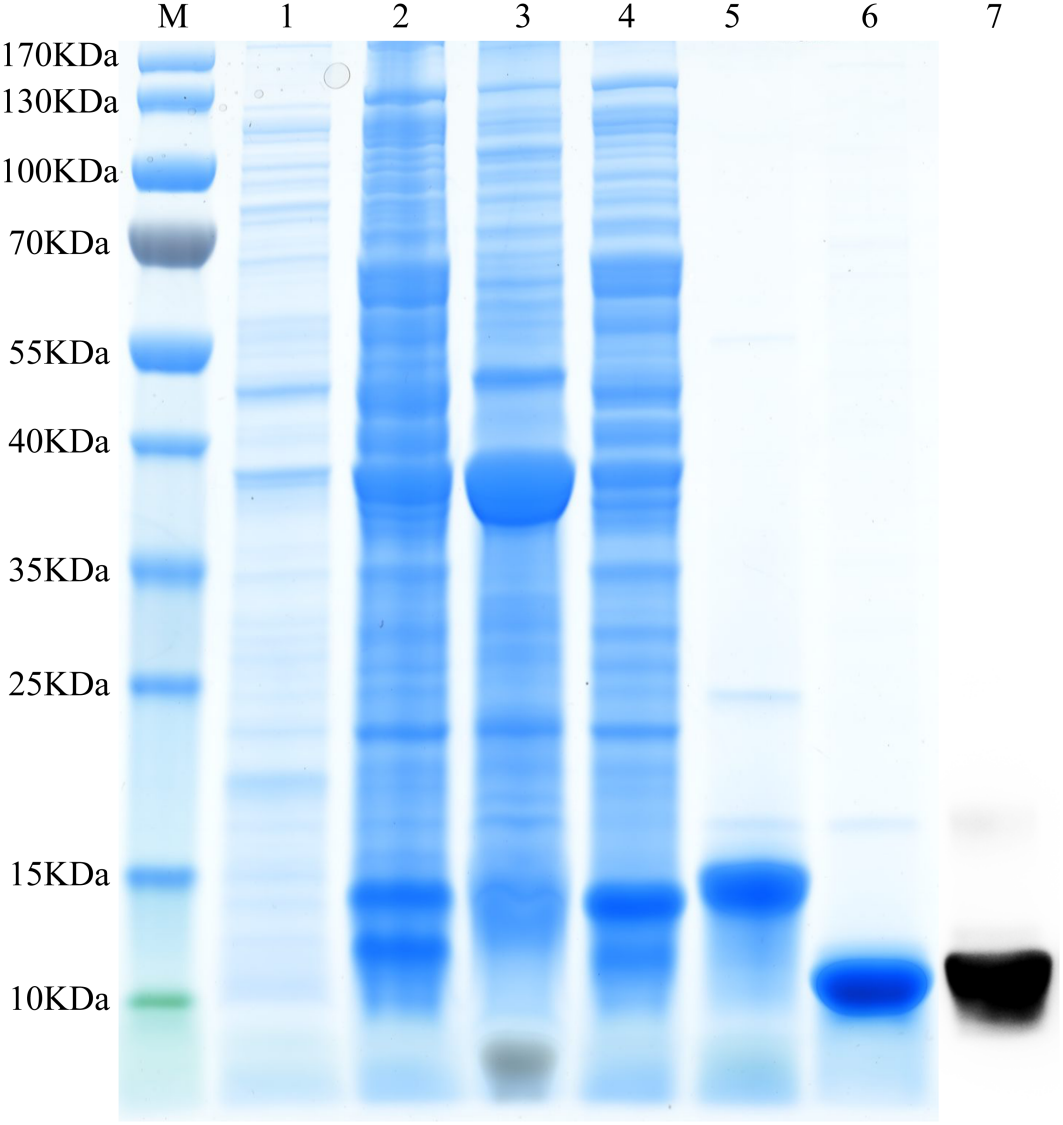
SDS-PAGE and western blot analysis of recombinant MmedCSP3. M: Molecular weight marker; 1: Non-induced pET-30a (+) / MmedCSP3; 2: Induced pET-30a (+) / MmedCSP3; 3: pET-30a (+) / MmedCSP3 supernatant; 4: pET-30a (+) / MmedCSP3 pellet; 5: Purified MmedCSP3 with His-tag; 6: Purified MmedCSP3 without His-tag; 7: Western blot analysis of MmedCSP3.

**Fig 5:**
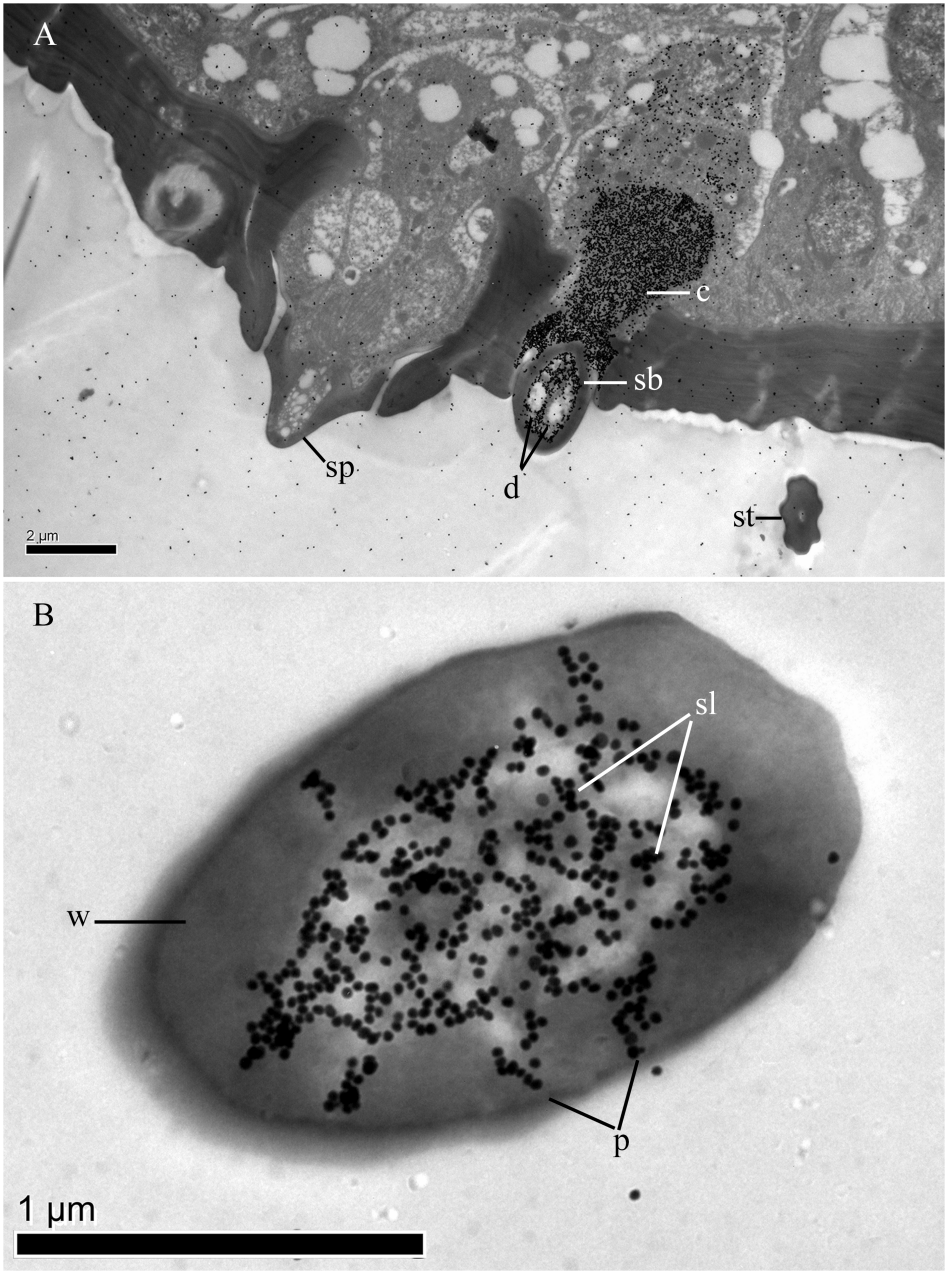
Immunocytochemical localization of MmedCSP3 in sensilla on female antennae. (A): The sensilla basiconica type 2 was labeled strongly, whereas the sensilla placodea were not labeled. (B): The sensillum lymph in the sensilla basiconica type 2 was strong labeled. sp, s. placodea; st, s. trichodea; c, cavity; d, dendrites; p, pore; w, sensillum wall.

Focusing on MmedCSP3, the anti-MmedCSP3 antiserums were used to mark the cellular localization of MmedCSP3 in antennal sensilla of *M*. *mediator*. Western blot analysis showed that the antibody could specifically bind to the purified recombinant MmedCSP3 (Fig 4). Immunolocalization results indicated that MmedCSP3 could be labeled in the sensilla basiconca type 2 (Fig 5A and 5B). The gold granules were concentrated at the sensillum lymph in the sensillum hair lumen and the cavity below the hair base. However, there was no MmedCSP3 labeled in the sensilla placodea. Immunolocalization data of MmedCSP3 in antennal sensilla of males were consistent with that in female antennae.

### Fluorescence binding assay

**Fig 6:**
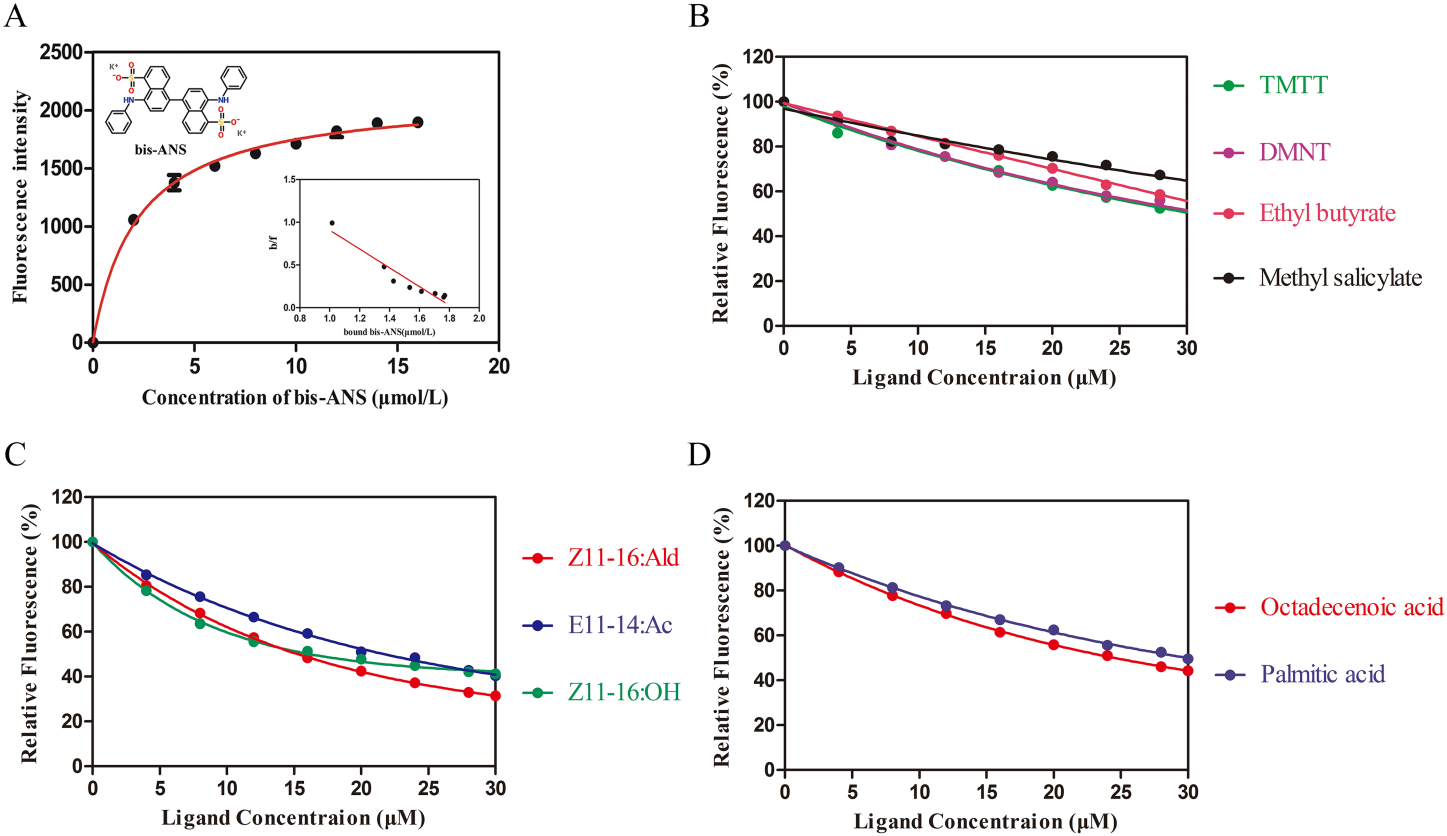
Binding characteristic of selected ligands to MmedCSP3. (A) Binding curve and relative Scatchard plot of bis-ANS to MmedCSP3. (B) Competitive binding curves of four selected plant volatiles to MmedCSP3 (C) Competitive binding curves of three sex pheromone components to MmedCSP3. (D) Competitive binding curved of two fatty acids to MmedCSP3.

The relative affinities of the recombinant MmedCSP3 with 102 candidate compounds were evaluated by fluorescence binding assays (Table 3). The candidate compounds were selected from known plant volatiles and some specific host insect odorants. The dissociation constant of the MmedCSP3 / bis-ANS complexes was 1.75 ± 0.42 μM (Fig 6A). Among the tested compounds, only nine chemicals showed certain affinities with MmedCSP3. The selected plant volatiles, (3*E*, 7*E*)-4, 8, 12-trimethyltrideca-1, 3, 7, 11-tetraene (TMTT), 1, 1-dimethyl-2-[4-(5-nitrofuran-2-yl)-1,3-thiazol-2-yl] hydrazine (DMNT), ethyl butyrate, and medthyl salicylate exhibited relatively weak binding abilities with MmedCSP3, and the Ki values ranged from 26.79 μM to 39.22 μM (Fig 6B). Interestingly, octadecenoic acid, palmitic acid, three sex pheromone components of Noctuidae insects, *cis*-11-hexadecenyl aldehyde (*Z*11-16: Ald), *cis*-11-hexadecanol (*Z*11-16: OH), and *trans*-11-tetradecenyl acetate (*E*11-14: Ac), displayed high binding abilities, and the Ki values ranged from 17.24 μM to 22.20 μM (Fig 6C and 6D).

**Table 3:** Binding affinities of selected ligands with recombinant MmedCSP3.

Ligands	CAS Number	Ki (μM)
*cis*-11-hexadecanol ^a^	56683-54-6	17.55 ± 1.19
*cis*-11-hexadecenyl Aldehyde ^a^	53939-28-9	17.24 ± 1.67
*trans*-11-tetradecenyl acetate ^a^	33189-72-9	18.77 ± 2.06
Ethyl butyrate ^b^	105-54-4	32.87 ± 7.43
Methyl salicylate ^b^	119-36-8	39.22 ± 0.25
TMTT ^b^	62235-06-7	26.79 ± 1.27
DMNT ^b^	26049-69-4	28.41 ± 1.05
Octadecenoic acid ^c^	112-80-1	18.21 ± 1.74
Palmitic acid ^c^	57-10-3	22.2 ± 1.82

Note:

^*a*^, sex pheromone components of Noctuidae insects.

^*b*^, plant volatiles

^*c*^, volatiles of both plants and insects.

Ki, dissociation constant, affinities (mean ± SD) measured in μM.

More potential ligands were tested, but the remaining 93 potential ligands did not bind the MmedCSP3. These compounds were 12-bromododecan-1-ol, 1,8-cineole, carveol, citronellol, (*E*, *E*)-farnesol, geraniol, hexanol, 2-hexanol, 3-hexanol, (*E*)-2-hexen-1-ol, (*Z*)-3-hexen-1-ol, (*E*)-3-hexen-1-ol, isooctan-1-ol, linalool, nerol, nerolidol, 1-octanol, pentanol, phytol, *cis*-9-tetradecenyl acetate, benzaldehyde, citral, decanal, 3,4-dimethyl-benzaldehyde, 4-ethylbenzaldehyde, hexanal, (*E*)-2-hexen-1-al, *cis*-9-hexadecenal, heptanal, (*Z*)-9-tetradecenal, nonanal, octanal, valeric aldehyde, amyl butyrate, butyl butyrate, butyl formate, *cis*-5-decenylacetate, (*Z*)-8-dodecenyl acetate, (*Z*)-7-dodecenyl acetate, ethyl caprylate, ethyl heptanoate, ethyl laurate, ethyl phenylacetate, geranyl acetate, heptyl acetate, hexyl hexanoate, (*Z*)-11-hexadecenyl acetate, hexyl acetate, (*Z*)-3-hexenyl acetate, (*Z*)-3-hexenyl hexanoate, (*Z*)-3-hexenyl butyrate, (*E*)-2-hexenyl butyrate, linalyl acetate, methyl jasmonate, methyl phenylacetate, nonanyl acetate, octadecyl acetate, octyl acetate, pentyl acetate, 2-phenylethyl acetate, *cis*-9-tetradecenyl acetate, (*Z*, *E*)-9, 11-tetradecadienyl acetate, (*Z*, *E*)-9, 12-tetradecenyl acetate, 2-heptanone, 2-hexanone, 3-hexanone, β-ionone, 2-nonanone, 2-octanone, carvacrol, indole, 2-methylnaphthalene, naphthalene, m-xylene, camphene, (1*S*)-(+)-3-carene, β-caryophyllene, α-farnesene, α-humulene, (*S*)-(-)-limonene, myrcene, β-ocimene, (-)-α-phellandrene, β-pinene, α-terpinene, terpinolene, decane, dodecane, octane, tridecane, undecane, linolic acid, and stearic acid.

## Discussion

In the present study, we obtained 20 OBPs and 3 CSPs from the antennal transcriptome of *M*. *mediator*, among them 10 OBPs and 2 CSPs were newly reported. There are 52 OBPs and 4 CSPs identified in *Drosophila melanogaster* [34], 45 OBPs and 16 CSPs in *Bombyx mori* [35], while *Apis mellifera* has 21 OBPs and 6 CSPs [15, 27], *C*. *cunea* has 25 OBPs and 11 CSPs [28]. The total number of OBPs and CSPs in *M*. *mediator* is smaller than in *D*. *melanogaster* and *B*. *mori*, but similar to those in *A*. *mellifera* and *C*. *cunea*. We suspected that there was less OBPs and CSPs in Hymenoptera insects. Of course, some potential OBPs and CSPs may not able to be identified in *M*. *mediator* due to low expressions level in the antennae or specific expression in other tissues.

Both *MmedOBP1–10* and *MmedCSP1* have been reported highly abundant or specific in the antennae of *M*. *mediator* [19, 20]. In the current study, *MmedOBP14*, *MmedOBP18* and *MmedCSP2–3* were abundantly expressed in the antennae of wasps. Antennae-enriched odor carrier proteins of *M*. *mediator* play important roles in detecting mates and locating suitable hosts. In insects, most OBPs are specifically or mainly expressed in chemosensory organs, whereas some OBPs are also expressed in non-chemosensory organs suggesting their potential non-olfactory functions. For example, *MmedOBP20* was highly expressed in abdomen, which might be involved in the physiological process of reproduction. Generally, CSPs are considered to be widely distributed throughout the body of insects. However, antenna-specific CSPs were also reported in the *Adelphocoris lineolatus* [16, 33]. *MmedCSP2–3* were mainly expressed in antennae of both sexes suggesting important roles in chemoreception of *M*. *mediator*.

There are six types of sensilla including sensilla basiconica identified in antennae of *M*. *mediator* [36, 37]. Sensilla basiconica are multiporous chemosensilla [38]. MmedCSP3 had high level in sensillar lymph of sensilla basiconica type 2 indicating its roles in olfactory perception of *M*. *mediator*. In fluorescence competitive binding assays, MmedOBP3 showed certain binding affinities with selected plant volatiles. TMTT and methyl salicylate are components of herbivore induced plant volatiles (HIPVs), which are considered dependable cues for foraging parasitoids [39, 40]. Palmitic acid and octadecenoic acid are long-chain free fatty acids, which are commonly found both in plants and insects [41]. It was demonstrated that female *Cotesia glomerata* could use fatty acids from host-damaged leaves to search host insects [42]. MmedOBP3 of *M*. *mediator* may play important roles in detecting plant volatiles, and we proposed that *M*. *mediator* could use plant volatiles or insect odors to locate hosts or sexual partners. Two semiochemicals, *Z*11-16: Ald and *Z*11-16: OH are main sex pheromone components of cotton bollworm [43, 44], *E*11-14: Ac is a sex pheromone component of *Spodoptera litura* [45]. Interestingly, in the present study, MmedCSP3 displayed high binding affinities with these three chemicals. It was reported that *cotesia plutellae* could be attracted by the sex pheromones of its host, *plutella xylostella* [46]. MmedCSP3 may be involved in perception of host insect sex pheromones. *M*. *mediato*r could locate host insects by the detection of insect sex pheromones. Therefore, MmedCSP3 may play important roles in chemoreception of *M*. *mediator*. The MmedCSP3 could be used as a potential target to regulate the olfactory behavior of parasitic wasps. However, gene editing and behavioral assays need to be further performed to verify the roles of this protein.

## Supporting information

S1 Table The cycle threshold (CT) value of OBPs and CSPs in different tissues. (DOC)
