# Supplementary material for: Identification of odorant binding proteins and chemosensory proteins in Microplitis mediator as well as functional characterization of chemosensory protein 3
Source: PLoS One. 2017 Jul 21;12(7):e0180775. doi: 10.1371/journal.pone.0180775 (PMC5521769; doi:10.1371/journal.pone.0180775)
Supplement: S1 Table — (DOC) [file pone.0180775.s001.doc]

Table S1. The cycle threshold (CT) values of OBPs and CSPs in different tissues.

|  | Female antennas | Male antennas | Heads | Thoraxes | Abdomens | Legs | Wings |
| --- | --- | --- | --- | --- | --- | --- | --- |
| OBP11 | 28.207 | 29.885 | 15.598 | 14.627 | 16.8167 | 18.744 | 21.704 |
| OBP12 | 29.767 | 30.756 | 23.921 | 22.589 | 25.325 | 29.512 | 29.416 |
| OBP13 | 29.894 | 27.878 | 18.332 | 18.834 | 20.647 | 21.828 | 21.841 |
| OBP14 | 23.665 | 14.896 | 28.612 | 29.037 | 31.370 | 23.052 | 22.920 |
| OBP15 | 27.600 | 28.691 | 19.484 | 23.968 | 27.138 | 27.689 | 27.199 |
| OBP16 | 30.257 | 28.283 | 22.318 | 22.036 | 20.823 | 23.176 | 24.092 |
| OBP17 | 23.143 | 22.959 | 19.425 | 16.520 | 21.542 | 18.629 | 19.540 |
| OBP18 | 19.409 | 19.453 | 20.425 | 27.336 | 27.601 | 20.708 | 22.801 |
| OBP19 | 22.862 | 22.743 | 26.016 | 26.366 | 26.181 | 20.905 | 27.509 |
| OBP20 | 32.512 | 32.192 | 31.924 | 34.986 | 24.512 | 32.555 | 33.910 |
| CSP2 | 20.200 | 21.175 | 27.945 | 30.087 | 31.813 | 29.322 | 30.182 |
| CSP3 | 19.150 | 17.021 | 24.059 | 28.092 | 30.466 | 25.070 | 23.722 |
| β-actin | 19.787 | 20.179 | 19.658 | 19.897 | 20.235 | 19.907 | 20.558 |
